# Characterization and Arsenic Adsorption Behaviors of Water Treatment Residuals from Waterworks for Iron and Manganese Removal

**DOI:** 10.3390/ijerph16244912

**Published:** 2019-12-05

**Authors:** Huiping Zeng, Tongda Qiao, Yunxin Zhao, Yaping Yu, Jie Zhang, Dong Li

**Affiliations:** Key Laboratory of Water Quality Science and Water Environment Recovery Engineering, Beijing University of Technology, Beijing 100124, China

**Keywords:** water treatment residuals, arsenic adsorption, iron and manganese removal

## Abstract

Water treatment residuals (WTRs), obtained from a groundwater treatment plant for biological iron and manganese removal, were investigated and used as adsorbents for arsenic removal. The surface morphology and structural features of the WTRs were characterized by scanning electron microscopy (SEM), transmission electron microscopy (TEM), X-ray diffraction (XRD), and Brunauner–Emmett–Teller analysis (BET). Laboratory experiments were also carried out to test the adsorption capability and adaptability of WTRs on both As (III) and As (V) removal from the water. The results showed that the WTRs were mainly amorphous and had a large specific surface area of 253.152 m^2^/g. The maximum adsorption capacities, evaluated using the Langmuir isotherm equation, were 36.53 mg/g and 40.37 mg/g for As (III) and As (V), respectively. The pseudo-second-order model fitted the kinetic data better, with R^2^ more than 0.99 for both As (III) and As (V). The removal of As (V) decreased with the increase in pH, especially when the pH was above 9, whereas for As (III), the removal effectiveness almost remained constant at both acidic and neutral pHs. H_2_PO_4_^−^ and SiO_3_^2−^ could strongly inhibit arsenic adsorption onto the WTRs, and the effect of other ions was little.

## 1. Introduction

Arsenic pollution in water is a global concern due to its toxicity and chronic effects on human health. Long-term exposure to arsenic-contaminated water increases the risk of the development of cardiovascular and hematological diseases, and cancers of the liver, lung, and skin [1]. The sources of arsenic in groundwater are from geochemical reactions, and industrial and agricultural activities. High concentrations of arsenic in groundwater have been widely reported all over the world, including in India, Bangladesh, China, Vietnam, and the United States. Taking into consideration the toxicology and cost of technology, the World Health Organization (WHO), the United States Environmental Protection Agency (USEPA), the Ministry of Health of People’s Republic of China (MHPRC), and the Bureau of Indian Standards (BIS) have decreased the maximum contaminant level (MCL) of arsenic in drinking water from 50 to 10 ug/L [2], creating a strong demand for economical and efficient treatment methods. 

Various arsenic-removal techniques have been developed, including precipitation, coagulation, membrane separation, ion exchange, lime softening and adsorption. Among these methods, adsorption and coagulation are the most promising and are widely used in the developing world [3], but the application of the coagulation method is limited, due to the requirement of skilled operators in small communities and at household levels. Solid adsorbents offer many advantages, including their simple operation and the easy handling of waste, and are suitable for use in rural remote areas. Among the solid adsorbents, iron-based substances are a promising arsenic adsorbent, due to their high specific surface area. These include granular ferric hydroxide, Fe-Mn binary oxide (FMBO) [4,5], magnetic binary oxide particles (MBOP) [6], ferrihydrite, goethite, nano-iron ion enrich material (NIIEM) [7], zero valent iron [8], natural hematite and natural siderite. However, many of these materials are not used in practical engineering applications because of their high price. Recently, researchers have paid more attention to the reuse of waste materials, such as red mud from the aluminum industry [9], blast furnace slag from the steel industry [10] and fly ash from thermal power plants [11], in order to not only reduce the cost of water treatment, but also supply methods for waste utilization. However, this waste requires chemical pretreatment, due to the presence of heavy metals. 

Compared with the three aforementioned wastes, Water Treatment Residuals (WTRs)—which are produced during the backwashing process from biofilters for iron and manganese removal in groundwater treatment plants—are the most promising, since they are not hazardous waste. At present, there are many biological iron and manganese removal water treatment plants in China, so large quantities of WTRs must be produced annually, and their treatment, disposal, and application need further research [12].

This study aimed to examine the adsorption behaviors of WTRs from waterworks for iron and manganese removal. More attention was paid to the characteristics of WTRs, and batch experiments were conducted to investigate the kinetics and isotherm characteristics of As (III) and As (V) adsorption onto WTRs.

## 2. Materials and Methods

### 2.1. Materials

**Figure 1:**
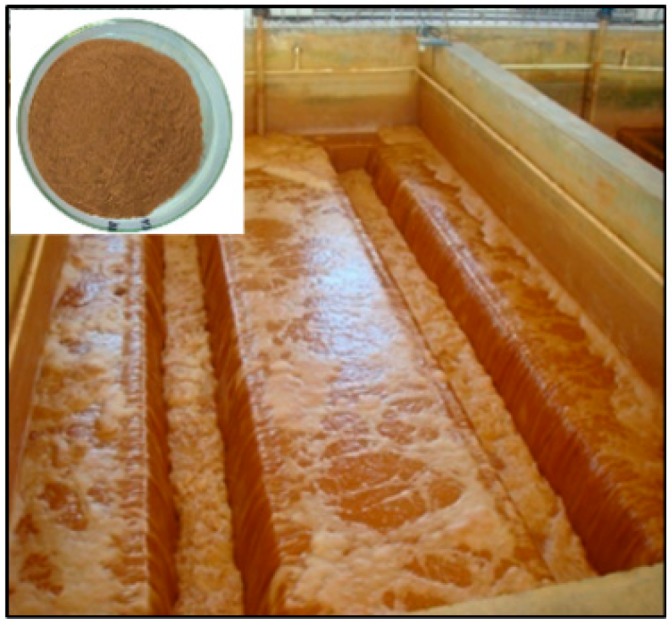
Backwashing water from the filter and water treatment residuals (WTRs) (inside picture).

WTRs (Figure 1) were taken from a biological groundwater treatment plant for iron and manganese removal using the aeration-biofiltration process, which was under steady operation for many years since its successful startup. The influent and effluent of the plant were 14.9 mg/L for Total Fe (TFe), 10 mg/L for Fe^2+^ and 0.8–1 mg/L for Mn^2+^, and 0.2 mg/L for TFe, 0.01 mg/L for Fe^2+^ and 0.02 mg/L for Mn^2+^. The backwashing water was collected and settled for several days, then thickened in the bottom, filtered with filter paper, and finally air-dried naturally. After that, it was crushed and sieved with 100 mesh screens to obtain a particle size under 147 um, then stored in a desiccator with blue silica gel particles for further analysis and experiments.

All chemicals used in this work were above analytical grade and purchased from Beijing Chemical Co. All solutions were prepared with deionized water. As (V) and As (III) stock solutions were prepared with disodium hydrogen arsenate Na_2_HAsO_4_·7H_2_O and sodium arsenite NaAsO_2_. The working solutions were prepared by diluting stock solutions with deionized water before use. NaOH and HCl with concentrations of 0.01 M, 0.1 M, and 1 M were used to adjust solution pH. Reaction vessels were cleaned with 1% HNO_3_ and rinsed several times with deionized water before use. Potassium borohydride and thiourea were of guarantee grade and solutions were prepared before use.

### 2.2. Characterization of WTRs

The samples of WTRs were characterized using the following method: microscopic examinations were conducted using a HITACHI S-4700 scanning electron microscope (SEM) with an Energy Dispersive Spectrometer (EDS) and a transmission electron microscope (TEM) (JEM 1200EX, Japan). The X-ray diffraction (XRD) pattern was recorded on a diffractometer (Bruker D8 Advance, Germany), using Co K_α_ radiation (l = 1.79026 A) at a 2θ range of 0–90°, and the operated current and voltage were 40 mA and 40 kV, respectively. The specific surface area of the WTRs was determined through nitrogen adsorption–desorption measurements (Micromeritics instrument corp, ASAP2460, USA). Chemical bond and functional group information was analyzed using a Fourier transform infrared spectrometer (Thermo Nicolet Corporation, Nicolet IS10, USA). The zero-charge point (pH_pzc_) was determined using the drift method [13]. A sample of WTRs (0.05 g) was added into 0.1 M NaNO_3_ solutions (100 mL) with different initial pH values (4.0–10.0) and reacted for 24 h, and the final pH values of solutions were tested. Then a curve correlated with the ΔpH and initial pH was plotted and the point where the curve crossed the axis determined the pH_pzc_ of the WTRs.

### 2.3. Bach Adsorption Experiments

To measure adsorption kinetics, 0.1 g of WTRs was added into polyethylene vessels containing 1000 mL solutions with 1 mg/L of initial arsenic (III) and arsenic (V) concentrations. The experiments were conducted at 25 °C on a mechanical orbit shaker at 100 rpm for 48 h. Samples were taken from the mixture at different time intervals.

In order to get more information about the kinetic characteristics of WTRs for adsorbing As (III) and As (V), to further understand the mechanism of the adsorption process, the research data were respectively fitted using the pseudo-first-order kinetic model and pseudo-second-order kinetic model given in Equation (1) and Equation (2).

(1) $$q_{t}=q_{e}\left(1-e^{-k_{1}t}\right)$$

(2) $$q_{t}=\frac{tk_{2}q_{e}^{2}}{1+k_{2}q_{e}t}$$

where *t* is the contact time of the adsorption test (h), *q*_e_ (mg/g) and *q*_t_ (mg/g) are the adsorption capacity at equilibrium status and at any time t, respectively, and *k*_1_ (1/h) and *k*_2_ (g/(mg·h)) are the rate constants of the two models, respectively. 

To measure the adsorption isotherms, 0.1 g of WTRs was added into stopper glass bottles containing 1000 mL As (V) solution and 500 mL As (III) solution, with different initial concentrations (from 0.1 mg/L to 50 mg/L) and a constant ionic strength of 0.01 M NaNO_3_. The samples were taken after 12 h of contact on a mechanical orbit shaker at 100 rpm for 12 h at 25 °C. 

To further understand the interaction between arsenic and WTRs, the data for the isotherm adsorption for As (III) and As (V) were fitted using the isotherm models of Langmuir and Freundlich given in Equation (3) and Equation (4).

(3) $$q_{e}=q_{m}\frac{K_{L}c_{e}}{1+K_{L}c_{e}}$$

(4) $$q_{e}=K_{F}c_{e}^{1/n}$$

where *q*_e_ (mg/g) is the As concentration adsorbed on the WTRs, *q*_m_ (mg/g) is the maximum adsorption capacity of WTRs, *c*_e_ (mg/L) is the equilibrium As concentration in solution, *K*_L_ (L/mg) is a constant related to the affinity of binding sites, *K*_F_ (mg/g) is a rough indicator of the adsorption capacity, and 1/*n* is a heterogeneity factor.

To measure the influence of the initial solution pH, a series of stoppered conical flasks containing 1000 mL of 1mg/L As (V) solution were prepared and 0.1 g of WTRs was added into every flask. Also, flasks containing 500 mL of 2 mg/L As (III) were prepared and 0.2 g of WTRs was added. The initial pH was adjusted in the range of 3.0–11.0 by adding solutions of NaOH or HCl. The samples were taken after 12 h of contact on a mechanical orbit shaker at 100 rpm for 12 h at 25 °C. 

The effects of common coexisting anions (SO_4_^2−^, HCO_3_^−^, SiO_3_^2−^ and H_2_PO_4_^−^) were also tested. A series of stoppered conical flasks containing 1000 mL of 1 mg/L As (V) solution was prepared and 0.5 g of WTRs was added into every flask. Also, flasks containing 500 mL of 2 mg/L As (III) were prepared and 0.5 g of WTRs was added. The specified concentrations of each coexisting ion were controlled at 0.1, 1, and 10 mmol/L. The samples were taken after 12 h of contact on a mechanical orbit shaker at 100 rpm for 12 h at 25 °C. All water samples were filtered using a 0.45 um membrane before test. Three parallel tests were conducted for each test, and the average value was taken for analysis.

### 2.4. Analytical Methods

The residual Arsenic concentrations were measured using an atomic fluorescence spectrophotometer (AFS-8230, Beijing Jitian instrument Co. Ltd.). Three parallel aqueous samples were prepared and an average of these three measured Arsenic concentration values was used for analysis.

## 3. Result and Discussion

### 3.1. WTRs Characterization

**Figure 2:**
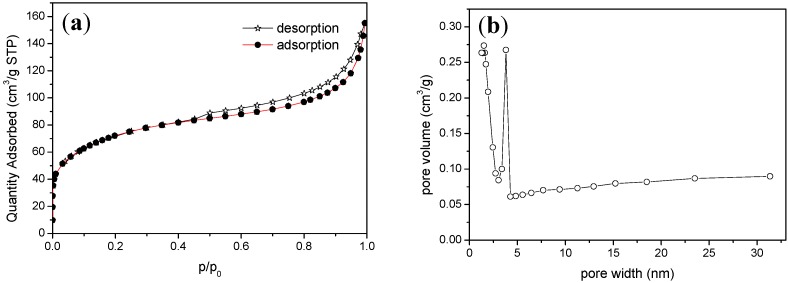
(**a**) N_2_ adsorption and desorption isotherms and (**b**) pore size distribution of WTRs.

The specific surface area of WTRs was determined using the nitrogen adsorption method (Figure 2a,b). It was 253.152 m^2^/g according to the Brunauner–Emmett–Teller (BET) analysis model, and such a large specific surface area is particularly conducive to adsorption. On the basis of International Union of Pure and Applied Chemistry (IUPAC) classification, the adsorption isotherm of WTRs was almost type Ⅳ with hysteresis loop type H3, having adsorption curve characteristics of typical mesoporous materials. The BJH desorption cumulative volume of pores between 1 nm and 300 nm diameter was 0.2199 cm^3^/g, and the BJH desorption average pore diameter was 3.6914 nm. 

**Figure 3:**
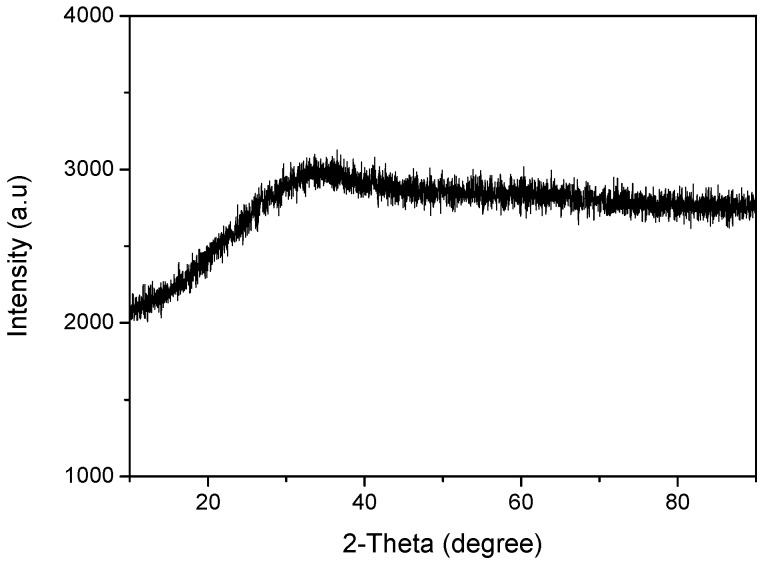
X-ray diffraction (XRD) pattern of WTRs.

The previous research showed that iron oxides are easy to form by the oxidation of Fe^2+^ in natural water [14], but other ions such as silicic acid, calcium and manganese also existed in the groundwater, which were doped in the iron oxides, hindering the crystallization of hydrous iron oxide, and the γ-FeOOH formed did not have the complete crystal structure that could be detected by X-ray, but has the finer γ-FeOOH under the detection limit [14]. The results of this research (Figure 3) also showed that the WTRs were amorphous, which is beneficial for the arsenic removal, because the amorphous structure greatly increases the surface areas of WTRs, as well as active sites. This is consistent with the previous results of BET.

**Figure 4:**
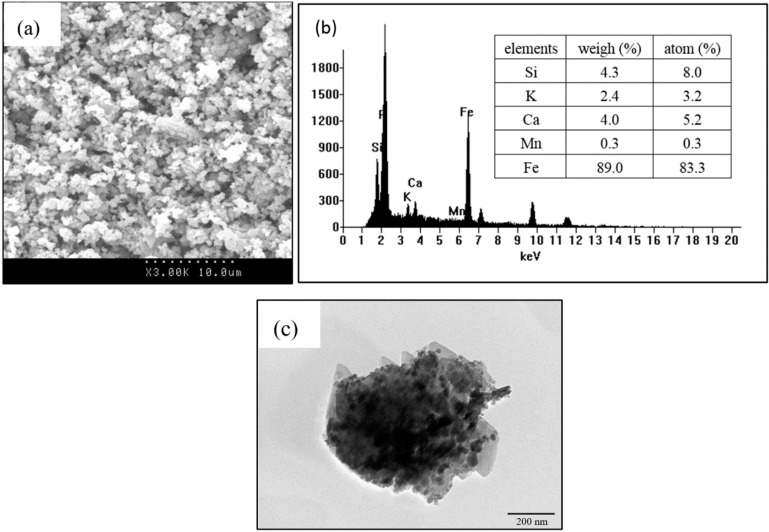
Images of SEM (**a**) with EDS spectra (**b**) and TEM (**c**) of WTRs.

SEM was an efficient method to check the surface morphology of WTRs. It can be seen in Figure 4a that the WTRs have a lot of aggregated small particles, forming a rough surface with a porous structure. In order to confirm the small particles, TEM analysis (Figure 4c) was carried out, revealing flaky and fine spherical structures. The EDS results (Figure 4b) indicated that Fe was the main element in the WTRs, and that Mn, Si, K, and Ca were also detected. These were the impurities as mentioned earlier in the analysis of the X-ray diffraction (XRD).

Experiments showed that the pH_pzc_ of the WTRs was 6.7, so the surface of WTRs could exhibit a positive or a negative charge. When the pH of the solution was below or above 6.7, it greatly influenced the reaction between WTRs and arsenic [15].

### 3.2. Adsorption Kinetics

**Figure 5:**
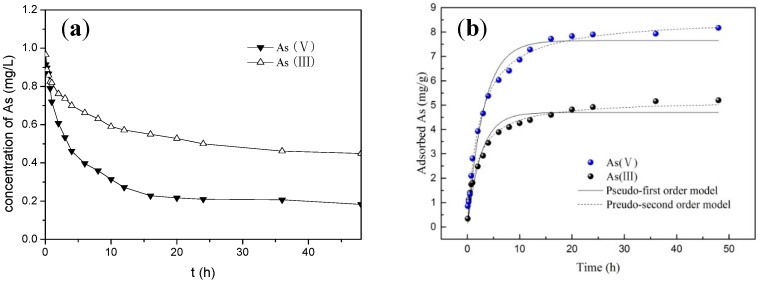
Curves of residual concentration (**a**) and adsorption kinetics (**b**) of As (III) and As (V) (the solid to liquid ratio is 0.1 g/L).

As we know, there are usually three stages during the adsorption process for the porous adsorbent [16], and this experiment obtained a similar result, as shown in Figure 5. For the first stage, of 0–8 h—presumed to be the film diffusion—the adsorbate diffused to the liquid film of adsorbent surface. The adsorption process was very quick, due to the adsorption sites on the surface and the concentration differences both being sufficient. For the second stage of 9–20 h—presumed to be the internal diffusion—when the surface adsorption was saturated, it would gradually diffuse to the interior, and the adsorption rate gradually decreased with the increase in diffusion resistance. Finally, in the third stage of 21–48 h, the adsorption process eventually reached equilibrium.

It can be seen from Figure 5a, that the final concentration of As (III) and As (V) was above 0.18 mg/L, which is not as good as similar experiments achieved in previous studies [15], exceeding the maximum contaminant level (MLC) of 10 ug/L for arsenic in drinking water. The reason should be that the ratio of solid-to-liquid in this research was 0.1 g/L, a much smaller value when compared with 2 g/L. But, for this reason, after reacting for 48 h, a bigger equilibrium adsorption amount of 8.17 mg/g was obtained in comparison to the corresponding value in previous studies (less than 1 mg/g). For the latter situation, the large ratio of solid-to-liquid used means that the adsorbents are adequate but the adsorbates are insufficient. 

The adsorption data of both As (III) and As (V) were fitted better (Figure 5b) using a pseudo-second-order kinetic model than using a pseudo-first-order kinetic model, with the correlation coefficients R^2^ 0.992 and 0.998 compared with 0.985 and 0.887, respectively. The results indicate that there was a chemical mechanism between arsenic and the WTRs during the adsorption process.

### 3.3. Adsorption Isotherms

**Figure 6:**
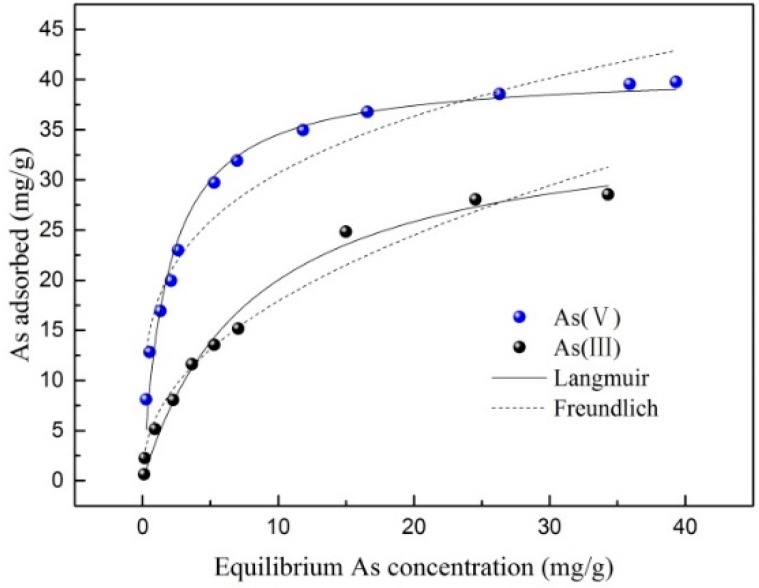
Adsorption isothermal of As (III) and As (V).

As can be seen from Figure 6 and Table 1, the Langmuir model fitted the adsorption process of As (III) and As (V) little better than the Freundlich model, and the R^2^ of the Langmuir model was 0.982 and 0.994 for As (III) and As (V), respectively, compared with results of 0.974 and 0.973 in the Freundlich model for As (III) and As (V), respectively. Actually, the value of R^2^ for both models was high. The adsorption could be fitted by both models. 

The maximum adsorption capacities calculated by the Langmuir model were 36.53 mg/g and 40.37 mg/g for As (III) and As (V), respectively. This revealed that the studied WTRs showed very high maximum adsorption capacities compared with other iron and manganese oxide adsorbents (Table 2). The As (V) removal ability is similar to other WTRs, which are much stronger than other kinds of adsorbents. At the same time, the WTRs used in this test had a strong removal capacity of As (III) of about 36.53 mg/g, which is much greater than that of WTRs consisting of amorphous Al/Fe oxide (15 mg/L), probably due to their containing manganese oxide, which has As (III) oxidation capacity [4].

### 3.4. Influence of Initial pH of Solution and Coexisting Anions 

**Figure 7:**
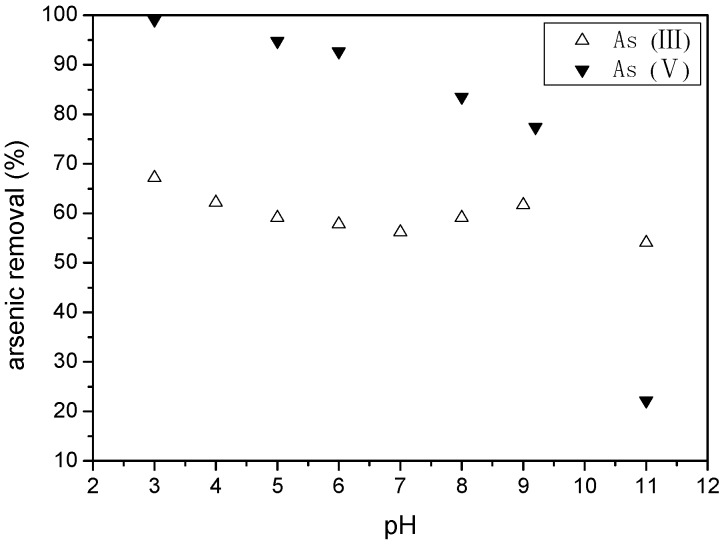
Effect of pH on arsenic removal by WTRs.

pH is important in the adsorption process, and the results about the effect of initial solution pH on arsenic removal are as follows (Figure 7): for arsenate, there was a little decrease from almost 99.12% to 92.67%, under the pH from 3 to 6, and it kept on decreasing little by little until it had a pH near 9, but when the pH reached 11, the removal rate had a dramatic reduction to about 22.19%. The reason was given as being that, firstly, hydroxyl ions increased at a pH of 11, and it enhanced the competition with As (V). Secondly, As (V) changed to more negative ions from H_2_AsO_4_^−^ to HAsO_4_^2−^ and AsO_4_^3−^, which made the electrostatic repulsion between arsenate and WTRs (negative charge under the condition of pH > 6.7) bigger. However, for arsenite, the influence was little, not only in the acidic and neutral environments, but also in the alkaline environment, and it remained at about 60% during the whole experiment. Firstly, in the acidic condition, due to the form of undissociated H_3_AsO_3_, the adsorption of arsenite on iron oxides did not change a lot and it was ineffective compared with arsenate. Secondly, in the alkaline environment, the reason why the removal rate did not decrease a lot like arsenate was that there might be some manganese dioxide in the WTRs, so it could oxidize As (III) to As (V) and result in the release of Mn^2+^ cations, which then could be adsorbed on the WTRs, give a positive charge and benefit the adsorption of arsenic [5].

In the natural water environment, many anions such as SO_4_^2−^, HCO_3_^−^, SiO_3_^2−^ and H_2_PO_4_^−^ often coexist with arsenic. Furthermore, the effect of these anions was as follows: the removal rate was more or less not affected by SO_4_^2−^. For SiO_3_^2−^ and H_2_PO_4_^−^, they caused a big drop of 7.1% and 11.4% for As (III), and 27.4% and 32.1% for As (V) during the range of anion concentration from 0.1 mmol/L to 1 mmol/L, because these two anions could form an inner-sphere complex with iron oxide, and compete with arsenic anions. In particular, the molecular structure of H_2_PO_4_^−^ was very similar with arsenate and arsenite, because As and P elements are in the same main group in the periodic table. However, for HCO_3_^−^, though it could form an inner complex with iron oxide, and the increase in pH value caused by HCO_3_^−^ in the water could enhance the electrostatic repulsion between As anions and WTRs, the removal rate did not decrease so much, ranging from 91% to 79%, then 58% for As (III), and from 73% to 63%, then 55% for As (V), with the concentration of HCO_3_^−^ ranging from 0.1 mmol/L to 1 mmol/L, then 10 mmol/L. The main reason is that the shared charge of As was smaller than that of HCO_3_^−^, which made the WTRs have a stronger affinity with As over HCO_3_^−^ [22].

### 3.5. Possible Mechanisms for Arsenic Adsorption

**Figure 8:**
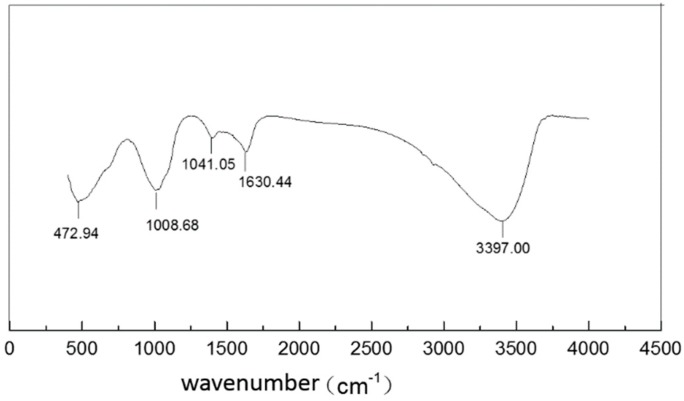
FTIR of WTRs.

It can be seen from FTIR (Figure 8) that WTRs contain a lot of –OH, which may be an important factor for arsenic removal. In much of the research on the mechanisms of arsenic removal by iron oxides, it is generally believed that the complexation adsorption mode is the main model, and the hydroxyl structure on the surface of iron oxides participates in arsenic adsorption and forms Fe-O-As [23].

Up to now, it was thought that this arsenic adsorption by WTRs included two mechanisms: physical adsorption and chemical specific adsorption. Their large specific surface area and large amount of –OH are the most important reasons for the strong arsenic adsorption capacity of WTRs.

## 4. Conclusions

WTRs—by products of the groundwater treatment plant for biological iron and manganese removal—were used as arsenic adsorbents from a water solution. The characterization of WTRs was conducted and laboratory experiments were also carried out to evaluate their arsenic adsorption behaviors. With maximum adsorptions of 36.53 mg/g and 40.37 mg/g for As (III) and As (V), respectively, WTRs should be considered as a promising low-cost adsorbent for the purification of arsenic-containing water solutions, and this would also provide a novel and feasible solution for the treatment and disposal of backwashing sludge wastes from groundwater treatment plants for iron and manganese removal. 

## Figures and Tables

**Table 1:** Parameters of the Freundlich and Langmuir isotherm model.

	Langmuir			Freundlich		
	Q_m_(mg/g)	K_L_(L/mg)	R^2^	1/n	K_F_(mg/g)	R^2^
As (III)	36.525	0.120	0.982	0.437	6.615	0.974
As (V)	40.372	0.528	0.994	0.271	15.964	0.973

**Table 2:** Comparison of the maximum adsorption capacities of different adsorbents.

Adsorbent	pH	q_max_ (mg/g)		References
		As (III)	As (V)	
Water treatment residual (Fe/Al/Mn)	7.2	-	3.3–50	[17]
Water treatment residual (Fe/Mn oxides)	8.1	-	42.9	[18]
Graphite oxide modified by Fe_3_O_4_ and MnO_2_	7.0	14.04	12.22	[19]
Blast furnace slag	12	1.4	-	[10]
Ferrous based red mud	7.25	0.9	-	[20]
WTRs (Amorphous Al/Fe oxide)	6.0–6.5	15	-	[21]
WTRs from waterworks for Fe and Mn removal	7.0	36.53	40.37	This study
